# Eyes on words: A fixation-related fMRI study of the left occipito-temporal cortex during self-paced silent reading of words and pseudowords

**DOI:** 10.1038/srep12686

**Published:** 2015-08-03

**Authors:** Sarah Schuster, Stefan Hawelka, Fabio Richlan, Philipp Ludersdorfer, Florian Hutzler

**Affiliations:** 1 Centre for Cognitive Neuroscience, University of Salzburg, Hellbrunnerstr. 34, 5020 Salzburg, Austria

## Abstract

The predominant finding of studies assessing the response of the left ventral occipito-temporal cortex (vOT) to familiar words and to unfamiliar, but pronounceable letter strings (pseudowords) is higher activation for pseudowords. One explanation for this finding is that readers *automatically* generate predictions about a letter string’s identity – pseudowords mismatch these predictions and the higher vOT activation is interpreted as reflecting the resultant *prediction errors*. The majority of studies, however, administered tasks which imposed demands above and beyond the intrinsic requirements of visual word recognition. The present study assessed the response of the left vOT to words and pseudowords by using the onset of the first fixation on a stimulus as time point for modeling the BOLD signal (fixation-related fMRI). This method allowed us to assess the neural correlates of self-paced silent reading with minimal task demands and natural exposure durations. In contrast to the predominantly reported higher vOT activation for pseudowords, we found higher activation for words. This finding is at odds with the expectation of higher vOT activation for pseudowords due to automatically generated predictions and the accompanying elevation of prediction errors. Our finding conforms to an alternative explanation which considers such top-down processing to be non-automatic and task-dependent.

The left ventral occipito-temporal cortex (vOT) plays a crucial role during unimpaired, proficient reading and is reliably activated by visually presented words or word-like stimuli^1^,^2^,^3^. The vOT is centered on the occipito-temporal sulcus lateral to the fusiform gyrus and extends from approximately *y* = −50 to *y* = −60 in Montreal Neurological Institute (MNI) space. Its function has often been studied by comparing its response to real words and pronounceable, but unfamiliar letter strings (i.e., pseudowords). This comparison predominantly revealed higher activation for pseudowords than for words^4^,^5^,^6^,^7^,^8^. An influential neurocognitive account of visual word recognition, that is, the Interactive Account^9^, interprets this finding as reflecting differences in the magnitude of *prediction errors*. Such errors are the consequence of a mismatch between a prediction regarding the identity of the perceived letter string and its actual identity. Predictions are thought to be generated in higher-order language areas (concerned, e.g., with phonology and semantics) and to propagate top-down to the left vOT which is considered as an interface integrating the top-down processing flow with the bottom-up information. The difference between bottom-up information and top-down prediction constitutes the prediction error. Critically, prediction errors are generally larger for unfamiliar letter strings (e.g., pseudowords) compared to previously encountered stimuli (i.e., familiar words) resulting in a higher activation of the left vOT^9^. However, while the general existence of top-down processes in visual word recognition is practically undisputed, their extent and automaticity is still a matter of debate^10^. To be specific, the explanation for the higher activation of the vOT for pseudowords as resulting from the *automatic* (i.e., task-independent and non-strategic) generation of predictions was questioned^11^. An alternative account proposes that the higher activation for pseudowords compared to words could be a consequence of (artificial) task demands and (inadequately) long exposure durations^11^. The present study investigated the response of the left vOT to words and pseudowords during self-paced, silent reading with a novel experimental technique, that is, fixation-related fMRI^12^,^13^ which allowed us to investigate left vOT activity with minimal task demands and natural exposure durations.

As aforementioned, the majority of studies report higher activation for pseudowords than words^14^,^15^,^16^,^17^,^18^,^19^. A few studies, however, found the opposite pattern^20^,^21^ or a similar activation for both types of stimuli^22^,^23^. A commonality of all studies is that the rate and duration of the stimulus presentation was fixed and predetermined (i.e., externally controlled). The specific timing of the rate and the duration, however, varied considerably *between* the studies. Of further theoretical importance is that the studies differed with respect to the requirements of the tasks – ranging from passive viewing to overt naming. To illustrate, the tasks which most consistently elicited higher activation for pseudowords than for words are the (phonological) lexical decision task and the (overt and covert) naming task^6^. The presentation durations in these studies typically ranged from 200 ms to 2000 ms. Evidently, these tasks impose – in comparison to natural (silent) reading – additional processing demands. The lexical decision task induces an exhaustive search of the mental lexicon (for rejecting pseudowords)^24^. The naming task requires phonological assembly^25^ (for a comparison of these two tasks see Ref. 22 and 6). In contrast, those studies which found a comparable activation for words and pseudowords administered implicit reading tasks coupled with a distractor task (e.g., hash-mark detection^23^,^26^) or a visual discrimination task (i.e., a brightness judgement^27^). The presentation durations in these studies were often substantially shorter than in the aforementioned studies which administered lexical decision and naming (e.g., 100 ms^23^,^26^). This brief sketch of the existing evidence underpins the notion that the activity of the left vOT seems highly susceptible to differences in task demands and exposure duration^11^; a notion recently confirmed by studies which directly compared the activation pattern of the left vOT during different tasks^27^,^28^,^29^,^30^. The fact that the difference in the activation of the vOT during the processing of words and pseudowords is modulated by the properties of the experimental task complicates the examination of the Interactive Account’s claim that the higher activation for pseudowords is due the automatic, task-independent generation of predictions and the resultant prediction errors.

Undoubtedly, all tasks have their merits, but for studying the automaticity (and the task-independence) of top-down processing in the left vOT a task would be required which, ideally, is devoid of any “surrogate” requirements above and beyond the mere processes of visual word recognition. A recent methodological add-on in the repertoire of fMRI techniques, that is, the fixation-related fMRI^12^, offers the possibility to present multiple stimuli simultaneously which, however, can be analyzed on the level of the individual items. This is achieved by the combined recording of blood-oxygen-level-dependent (BOLD) signals and eye movements. The onset of a first fixation on a stimulus is used as the time point for modeling the haemodynamic brain response. The method can provide several advantages compared to contemporary paradigms. Firstly, participants can process the presented stimuli at their own pace. Thus, the method provides “natural” presentation durations (i.e., the inter-individual and intra-individual fixation durations of the participants). Fixation durations reflect online cognitive processing such as the ease or difficulty of visual word recognition^31^. To exemplify, we expect longer fixation durations for pseudowords than words^32^,^33^. Secondly, and of particular relevance for the present study, the method abolishes the need of an additional (surrogate) task, because the eye movements serve as indicator for the engagement of the participants. Thus (and thirdly), the technique makes experimental settings possible which are ecologically more valid than traditional setups.

In a recent study, we proved the feasibility of the fixation-related fMRI technique for the domain of reading research^13^. This study simultaneously presented reading material (i.e., words and pseudowords) and non-reading material (i.e., slash-strings and strings of unfamiliar – i.e., Hebrew – letters). The stimuli were arranged in a circular array (for comparability with a previous study^12^) and the participants had to detect strings containing duplets of identical characters (e.g., Ho**bb**y, ℶחחדא). For the reading material, we obtained the typical activation in the left-lateralized reading network (i.e., within occipito-temporal, middle and superior temporal regions). Moreover, the technique proved to be sensitive enough to distinguish between the activation elicited by the different types of stimuli (pronounceable letter strings versus slash-strings and unfamiliar letters). The study, however, had a methodological focus providing the proof-of-concept for the applicability of fixation-related fMRI for reading research. The objective of the present study is to shed light on the hypothesized automaticity of predictions and resultant prediction errors when readers encounter pseudowords during natural (i.e., self-paced and silent) reading.

To recapitulate, from the perspective of the Interactive Account^9^ pseudowords should elicit higher activation in the left vOT than words, because the visual input (i.e., the bottom-up information) will deviate to a greater degree from the top-down generated predictions when readers encounter a pseudoword. As yet, the Interactive Account – postulating automatic and non-strategic top-down processing and the generation of predictions which result in prediction errors – is subject to an alternative explanation which ascribes the higher activations for pseudowords to *task-dependent* and *strategic* top-down processes. This task-induced and strategic top-down processing may be particularly pronounced in tasks with a sustained (i.e., too long and externally controlled) stimulus presentation and in tasks which require an overt response. By means of the novel fixation-related fMRI^12^,^13^, we assessed the response of the left vOT during the self-paced (i.e., internally controlled) processing of words and pseudowords without task-requirements above and beyond visual word recognition.

## Method

### Participants

Thirty-six undergraduate German speaking students (18 male) participated in the study (mean age: 25 years; *SD* = 4). All participants had normal or corrected-to-normal vision and reported no history of neurological or psychiatric disorders. Before scanning, participants gave their written informed consent. The experiment was conducted in accordance with the Code of Ethics of the World Medical Association (Declaration of Helsinki) and it was approved by the local ethics committee of the University of Salzburg (“Ethikkommission der Universität Salzburg”).

### Stimuli and Design

Stimuli were presented on a MR-compatible LCD screen (NordicNeuroLab, Bergen, Norway) with a resolution of 1024 × 768 pixels and a refresh rate of 60 Hz. The reading material consisted of 250 words and 250 pseudowords. Each stimulus consisted of 5 elements (i.e., letters or slashes) presented in a bold, monospaced font. The width of a single character corresponded to a visual angle of approx. 0.38° (string: ~1.9°). Words and pseudowords were rigorously matched (all *t*s < 1) on a substantial amount of influential sublexical variables. These variables encompassed number of syllables, orthographic Levenshtein distance^34^, OLD20 neighborhood *log*-frequency^34^ and various bigram frequency measures (i.e., mean *log*-frequency of all bigrams and of the initial and the final bigram). The *log*-frequency values of the words were derived from the *SUBTLEX* database^35^ and was on average 2.24 (*SD* = 0.72). Pseudowords were generated using the *Wuggy* software^36^.

**Figure 1:**
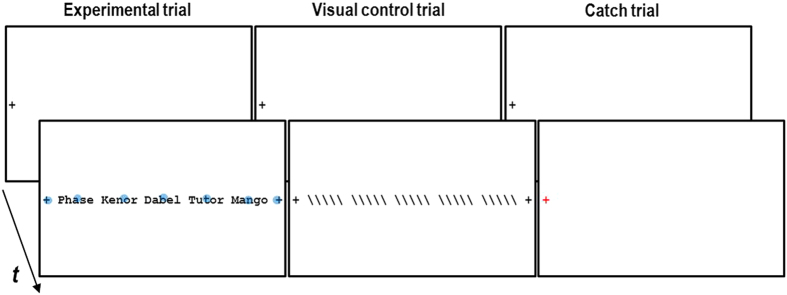
Experimental setup. After fixating the fixation cross at the left side of the screen, participants silently read the words and pseudowords or scanned the slash-strings. Fixating the cross at the right side of the screen terminated the trial. The red fixation cross of a catch trial required a button press within 2 sec. For the purpose of illustration, prototypical fixation locations are depicted in the experimental trial. The word (*w*) and pseudoword (*pw*) sequence in the example is *w*, *pw*, *pw*, *w*, *w*.

The experimental setup is depicted in Fig. 1. The experimental trials presented mixed lists of words and pseudowords (5 items per list). Before the presentation of a trial, a fixation-cross appeared at the vertical center of the left screen frame for a pseudo-randomly chosen duration (ranging from 1000 to 3000 ms with increments of 500 ms). While the participants fixated the fixation-cross, a drift correction or a fixation control was administered (see Eye Tracking section). Thereafter, the stimuli appeared which the participants read silently at their own pace. As visual control stimuli, we presented slash-strings (strings consisting of five backslashes) arranged in the same way as the words and pseudowords in the experimental trials which imposed comparable oculomotor demands. The participants were instructed to fixate each slash-string in a reading-like manner (“mindless reading”^37^). Fixating a cross near the vertical center of the right screen frame terminated a trial. In order to maintain the vigilance of the participants, interspersed catch-trials (14% of the trials) presented a red fixation cross and required a button press (time-limit: 2 sec). The presentation of the words (*w*) and pseudowords (*pw*) was counterbalanced with regard to a foveal and upcoming (i.e., parafoveal) item which resulted in a total of four different possible word/pseudoword sequences (i.e., *w w pw pw w, w pw pw w w, pw pw w w pw,* and *pw w w pw pw*). The different sequences were presented equally often (i.e., 25 times each) in a pseudo-randomized order. In sum, 100 trials presented words and pseudowords (i.e., a total of 250 *w* and 250 *pw*), 50 trials presented slashes (250 slashes), 30 were catch trials (i.e., red fixation cross) and 30 trials were null-events in which the (black) fixation-cross remained on the screen for 2 seconds.

### Data Acquisition and TreatmentEye-Tracking

Eye movements were recorded with a long-range *Eyelink CL system* (SR-Research, Ontario, Canada) with a sampling rate of 1 kH. The eye-tracker was placed at the end of the scanner bore. While recording monocular from the right eye, the head of the participants was stabilized in the head coil approximately 200 cm in front of the screen. The experiment was divided into three runs. Prior to each run the eye-tracker was calibrated with a horizontal three-point calibration. Each trial was preceded by a drift correction procedure to confirm the calibration parameters or a fixation control procedure in which a fixation had to be detected by the eye-tracking system around the fixation cross (40 × 40 pixels). In case the drift correction or the fixation control failed, the eye tracking system was re-calibrated. Note that half of the participants were tested using the drift correction, whereas the other half were tested using the fixation control. Fixations shorter than 80 ms and inaccurate eye movement measures (i.e., 3.5% of the data) were discarded from the analyses.

### fMRI

Functional imaging data were acquired with a Siemens Magnetom Trio 3 Tesla scanner (Siemens AG, Erlangen, Germany) equipped with a 12-channel head-coil. Functional images sensitive to blood-oxygen-level dependent (BOLD) contrast were acquired with a T2* weighted gradient echo EPI sequence (TR 2000 ms, TE 30 ms, matrix 64 × 64 mm, FOV 192 mm, flip angle 80°). Thirty-six slices with a slice thickness of 3 mm and a slice gap of 0.3 mm were acquired within the TR. The scan procedure encompassed 3 runs with a variable number of scans per run. The exact number of scans depended on the participants’ reading speed and the number of re-calibrations of the eye-tracking system and ranged from 149 to 494 scans (*M *= 205, *SD *= 50). In addition to the functional images, a gradient echo field map (TR 488 ms, TE 1* *= 4.49 ms, TE 2* *= 6.95 ms) and a high resolution (1 × 1 × 1.2 mm) structural scan with a T1 weighted MPRAGE sequence were acquired from each participant.

For preprocessing and statistical analysis, SPM8 software was used (http://www.fil.ion.ucl.ac.uk/spm/) running in a MATLAB 7.6 environment (Mathworks Inc., Natick MA, USA). Functional images were corrected for geometric distortions by the use of the FieldMap toolbox, realigned and unwarped, and then co-registered to the high resolution structural image. Note that due to technical issues, the correction for geometric distortions by means of the respective fieldmap was not viable for one subject. The structural image was normalized to the MNI T1 template image, and the resulting parameters were used for normalization of the functional images, which were resampled to isotropic 3 × 3 × 3 mm voxels and smoothed with a 6 mm FWHM Gaussian kernel. No slice timing correction was applied.

Statistical analysis was performed by means of computing a fixed effects model on the first level (i.e., single subject level) and a random effects model on the second level (i.e., group level). The eye-tracking data was related to the BOLD responses in the specifications of the first level model, that is, each first fixation on an item was modeled by a canonical haemodynamic response function combined with time and dispersion derivatives. The onsets of the catch-trials, which required a button press, were coded as a regressor of no interest. Furthermore, six movement parameters which were derived from the realignment preprocessing-step were modeled as covariates of no interest. The functional data in these first level models were high-pass filtered with a cut-off of 128 seconds and corrected for autocorrelation by an AR(1) model^38^. The parameter estimates of these first level models, reflecting signal change for words versus baseline (comprising the inter-stimulus intervals, the null-events, and the eye tracker drift correction/re-calibration procedures), pseudowords versus baseline, and slash-strings versus baseline were calculated in the context of a GLM^39^. This procedure resulted in subject-specific contrast images which were then used for the second level random effects analysis. In the second level analysis, significant effects on the whole-brain level were identified using a voxel-level threshold of *p* < .001 (uncorrected) and a cluster-level threshold of *p* < .05 (*FWE*-corrected for multiple comparisons).

## Results

### Behavioral results

The performance on the catch trials was at ceiling with a mean detection rate of 99.23%. As evident from Table 1, the words and pseudowords received, on average, a higher number of fixations than the slash-strings; main effect of stimulus type: *F*(2, 70) = 20, *p* < .01. Pairwise comparisons with a Wilcoxon ranked sum test revealed that the differences were significant; *W*s < 456, *p* < .05. The small difference in number of fixations between words and pseudowords was marginally significant; *W* = 703, *p* = .054. Moreover, the words received, on average, shorter first fixation and shorter gaze durations than pseudowords and slash-strings. The main effects of stimulus type were significant; *F*(2, 70) = 8.04, *p* < .01 and *F*(2, 70) = 5.30, *p* < .001 for first fixation and gaze duration, respectively. Pairwise comparisons revealed longer first fixation and gaze durations for pseudowords than words; *t*(35) = 3.79 and 5.73, respectively; *p*s < .001. The fixations times for pseudowords and slash strings did not differ, *t*s < 1.12.

### Fixation-related fMRI results

**Figure 2:**
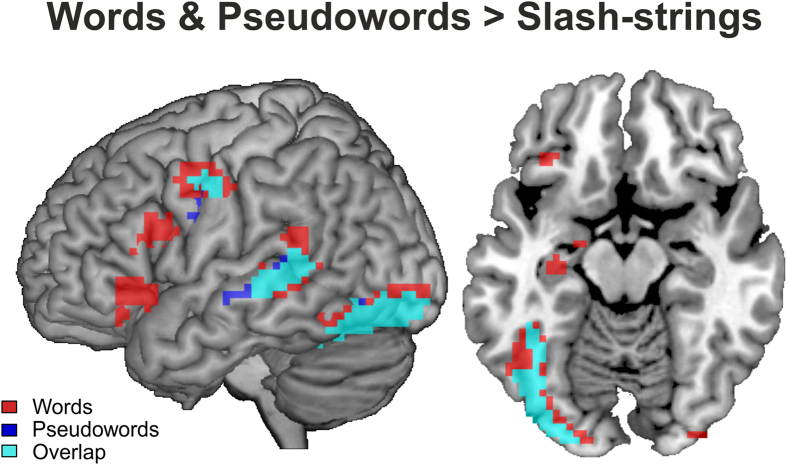
The surface rendering of the lateral and ventral (*z* = −14) views depicts the activation of words (red) and pseudowords (blue) contrasted against slash-string scanning. The overlap in the activation of words and pseudowords appears in cyan.

Figure 2 illustrates the results from separately contrasting the activation elicited by words and pseudowords against the activation elicited by slash-strings. Table 2 provides the respective peak voxels and the cluster extents of these contrasts. As evident from the Figure, words (red) and pseudowords (blue) activated similar brain regions (overlapping activation appears as cyan). To be specific, words and pseudowords elicited higher activation than the slash-stings in the left inferior occipital gyri from where the activation extended towards the anterior fusiform gyrus (including the visual word form area^1^,^2^,^3^,^40^,^41^; see Discussion). Furthermore, we observed activation in the left middle temporal gyrus and the left precentral gyrus. Words additionally activated the orbital as well as opercular parts of the left inferior frontal gyrus. This activation pattern conforms to the well-established, left-lateralized reading-network^4^,^5^,^6^,^7^,^8^.

Next we directly compared the activation for words compared to pseudowords (i.e., *w* > *pw* and *pw* > *w*). For the left ventral visual stream, the contrast words greater than pseudowords revealed differences in two clusters. One cluster was located in the posterior portion of the stream at *x* = −42, *y* = −61, *z* = −14 and had an extent of 9 voxels (peak *t*-value: 4.13). The other cluster was located in the anterior portion of the stream at *x* = −39, *y* = −46, *z* = −20 and had an extent of 8 voxels (peak *t*-value: 4.12). We note, however, that these differences were only reliable without cluster-level correction. The reverse contrast, that is pseudowords greater than words, did not reveal any significant differences within the left ventral visual stream.

**Figure 3:**
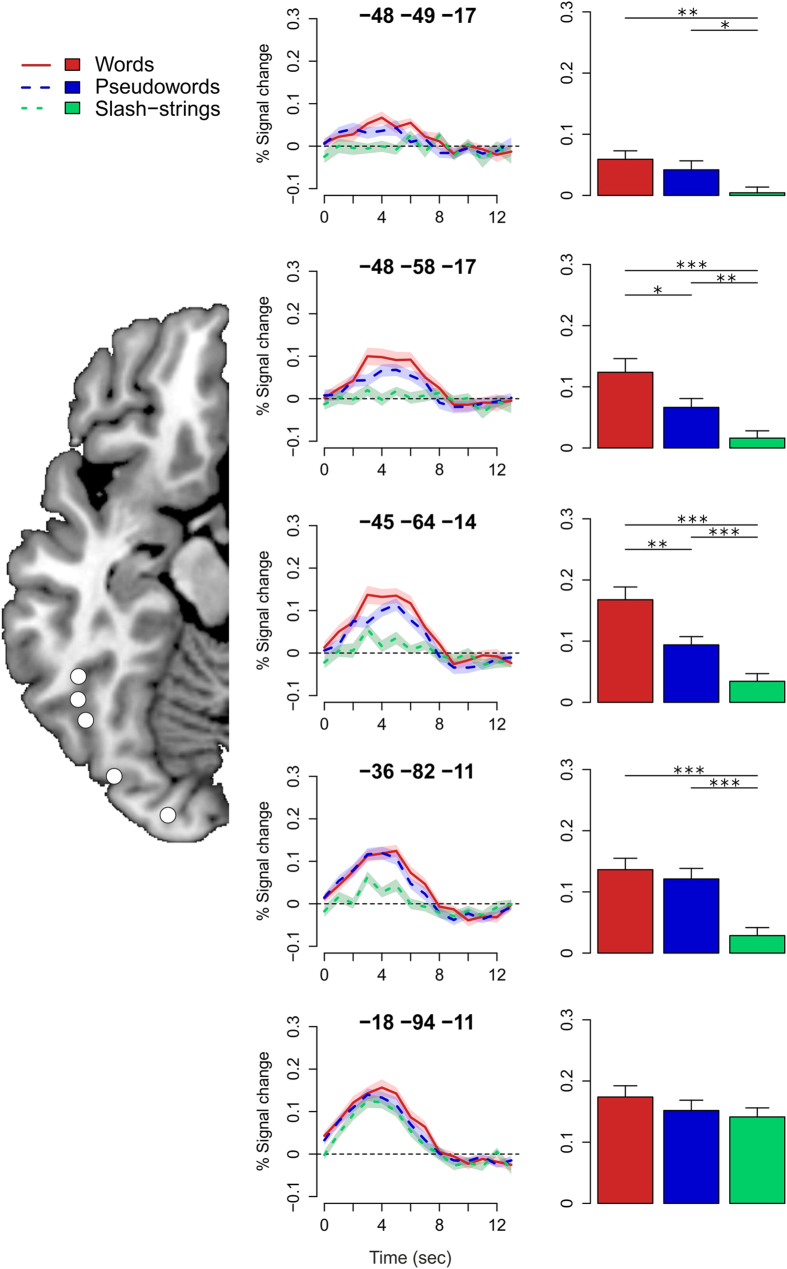
ROI analyses in left ventral visual stream. The left panel illustrates the location of the posterior-to-anterior ROIs. The middle column provides the *xyz*-coordinates of the ROIs and illustrates the event-related time course of the signal change for words, pseudowords and slash-strings. The polygons denote 1 standard error of the mean (SEM). The right column depicts the mean percent signal change (error bar = 1 SEM) with indications about significant differences between the words, pseudowords and the slash-strings: ***p < .001; **p < .01 and *p < .05.

We further investigated the difference between our types of stimuli by examining the activation pattern of the left ventral visual stream within several regions of interest (ROI). To this end, we selected five ROIs at posterior-to-anterior sites of the ventral stream according to the study of Vinckier and colleagues^23^. Each ROI was centered on activation maxima of an F-test representing the effects of interest with a maximum deviation of 3 mm from the original coordinates of Vinckier and colleagues. The resulting locations are denoted and illustrated in the middle and the left panel of Fig. 3, respectively. We obtained the event-related time course of the signal change and the signal change estimates (for spheres with a radius of 3 mm) with the MARSBAR toolbox^42^. The middle panel of Fig. 3 shows that the haemodynamic responses consistently peaked around approximately 4 seconds after fixation onset; their shape closely resemble those obtained by conventional event-related fMRI designs^43^. With regard to the differences in the activation for the different types of stimuli, the Figure shows that the slash-strings elicited the lowest activation, whereas the words elicited the highest activation throughout the posterior-to-anterior gradient of the left ventral visual stream. Analyses of variance revealed significant main effects of stimulus type for all, but the most posterior ROI with *F*(2, 70) = 2.55 (*p* = .09), and *F*s(2, 70) = 16.40, 22.84, 13.61 (*p*s < .001) and 4.32 (*p* < .05) posterior-to-anterior, respectively.

Next we administered pairwise comparisons for the ROIs for which the previous analyses revealed a significant main effect of stimulus type. We corrected the comparisons for multiple comparisons by means of the false discovery rate^44^. Significant differences in the activation for the different types of stimuli are denoted by the horizontal bars above the bar charts of Fig. 3. As evident from the Figure, the comparisons revealed significant differences for the words and the pseudowords compared to the slash-strings for each (except the most posterior) ROI; words vs slash-strings: *t*s(35) = 5.55, 6.71, 4.86 and 3.39; pseudowords vs slash-strings: *t*s = 4.54, 4.01, 2.94 and 2.36. The differences between words and pseudowords were not significant for the ROI at *y* = −82 and the most anterior ROI (*t*s < 1). For the ROIs at *y* = −64 and *y* = −58, the comparisons revealed significantly higher activation for words than pseudowords; *t*s = 3.13 and 2.58, respectively.

## Discussion

The present study assessed the activation of the left ventral occipito-temporal cortex (vOT) in response to unfamiliar (but pronounceable) letter strings (i.e., pseudowords) compared to familiar words. We found higher activation of the left vOT for words than pseudowords in two regions of interests (ROI). One of these ROIs is located in the left occipito-temporal sulcus, lateral to the fusiform gyrus; the other is slightly more posterior. Importantly, the former ROI closely corresponds to the location of the *visual word form area* (VWFA^1^,^2^,^3^; *x* = −45, *y* = −58, *z* = −11). In its original conceptualization, the function of the VWFA was attributed to the visual, bottom-up and prelexical analysis of words^3^,^40^,^41^. To be specific, the VWFA is considered to be sensitive to frequently recurring letter sequences such as bigrams and constitutes an integral part of a hierarchy of local combination detectors which are tuned – on a posterior to anterior gradient – to increasingly complex orthographic features (*cf*. the LCD model^40^). The Interactive Account reframed this bottom-up view by postulating an early influence on visual-orthographic processing by top-down *predictions* generated in higher-order language areas. Importantly, this account assumes that these predictions (and the resultant prediction errors) are generated automatically and task-independently^9^. Prediction errors are supposed to be greater for pseudowords — resulting in the typically reported higher activation for pseudowords than words in the left vOT.

Our result does not concur with this activation pattern predominantly reported in the literature^4^,^5^,^6^,^7^,^8^ and its neurocognitive explanation by the Interactive Account. To the contrary, our finding lends support to the explanation of the left vOT’s higher activation in response to pseudowords in terms of task properties (i.e., task demands and exposure durations). In the present study, task demands were “low” and “presentation durations” of the stimuli (if equated with fixation times) were short. Previous studies, which administered low-demanding tasks with short presentation durations, did not observe any activation differences for words and pseudowords^23^,^26^. To illustrate, Vinckier and colleagues^23^ assessed the left vOT response to words and pseudowords – presented for only 100 ms – while the participants were only required to detect interspersedly presented hash-mark strings. The communality of Vinckier *et al.*’s task and the silent reading task of our study is that it probably did not elicit (a too) deep processing of pseudowords (e.g., ruminating about its word-likeness). Many of the studies, which reported higher activation for pseudowords than for words, either administered the lexical decision task^16^,^19^,^21^ or a (covert or overt) naming task^14^ (reviewed in Ref. 6). For lexical decision, the multiple read-out model of visual word recognition^24^ postulates that participants have to perform an exhaustive search in the mental lexicon in order to reject a pseudoword. For naming a pseudoword, participants must – according to the dual-route cascaded model of reading aloud^25^ – assemble a phonological output representation from its grapheme-phoneme correspondences. The additional processing demands for pseudowords during lexical decision and naming, as theorized by the these computational models, may account for the higher activation for pseudowords than words in the left vOT.

Importantly, our finding of higher activation for words than for pseudowords, which does not conform to a main theoretical assumption of the Interactive Account, does not imply that visual word recognition is not an interactive process. In fact, there is a broad consensus that word recognition involves bottom-up as well as top-down processing^10^ which is best demonstrated by the word superiority effect^45^. This effect is explained by top-down processing from the whole-word level which influences bottom-up processing of the sublexical constituents of words. This mechanism accounts for the phenomenon that single letters are recognized better in real words than in consonant strings^45^. What our finding puts into perspective is the assumed automaticity of generating top-down predictions (about the identity of context-free, singly presented words) which the Interactive Account subsumed under the mechanisms of interactive bottom-up and top-down processing. Incorporating such a mechanism in an account of visual word recognition was inspired by the predictive coding framework of perception^46^. For object recognition, evidence accumulates that predictions about the probable identity of an perceived object facilitate recognition^47^,^48^. To be specific, visual information about the low-spatial frequencies of an object is projected extremely fast (via magnocellular pathways) to the orbito-frontal cortex^48^. From there, top-down propagated information (i.e., a “prediction” about the perceived object) facilitates its recognition by reducing the number of object representations which have to be considered^48^. Whether a similar mechanism could play a role for the (context-free) identification of written words (presented in isolation) is an open question. Importantly, in object recognition, predictive coding is influenced by context^49^. Thus, one may hypothesize that predictive coding in reading requires contextual information as well. Indeed, there is evidence that the depth of visually *pre*processing an upcoming word of a sentence depends on the constraints imposed by previous sentential context^50^,^51^,^52^.

We note, the design of the present study does not allow us to conclude upon the mechanism which may account for the higher activation of the left vOT for words than for pseudowords. As aforementioned, Dehaene, Cohen and colleagues^1^,^2^,^3^,^40^,^41^ considered the functioning of the VWFA to be prelexical in nature. From this perspective, one would expect a similar activation for words and well-matched pseudowords (in a task which emphasizes bottom-up processing by means of short presentation durations and low task demands^11^ – as it is the case in the present study). In the following we speculate about two potential underlying neurocognitive mechanisms which could account for the higher activation for words than for pseudowords. A first plausible explanation can be inferred from the *global workspace* theory which postulates that conscious perception (equated with word recognition in the present study) requires top-down amplification of neuronal activation in posterior brain regions from more anterior regions^53^. In our silent reading task (devoid of any demands except automatized word recognition), it might be that the prelexical analysis of words (in the sense of the LCD model^40^) activates the respective phonological (and/or semantic) representation in more anterior, higher-order language areas which, in turn, boosts the VWFA activation by means of top-down amplification.

A second explanation can be inferred from an alternative view on the function of the VWFA. It has been hypothesized that the function of the VWFA is not limited to the prelexical analysis of words, but that the area may act as a storage of orthographic representations of frequently encountered words (i.e., lexical processing)^54^. Evidence for this assumption is, firstly, that the VWFA does not exhibit reduced responsiveness (i.e., neural adaptation) for the subsequent presentation of orthographically highly similar words (e.g., COAT - BOAT)^55^. Secondly, electrophysiological (EEG) studies revealed that (high-frequency) words elicit a higher amplitude of an early component of the event-related potential (i.e., the N2 component) than pseudowords (or low-frequency words)^56^,^57^ in the left fusiform gyrus^57^. Thus, the higher activation for words could reflect the early instantiation of stored whole-word representations.

One may be apprehensive as to the validity of our results, because we used a very novel technique, that is, fixation-related fMRI. We made use of the technique, because it allowed us to investigate the processing of words and pseudowords during silent and self-paced reading – thereby abandoning two requirements of traditional fMRI experiments. First, fixation-related fMRI does not require fixed exposure durations and presentation rates and hence maintains the dynamics of natural reading: Participants are allowed to direct their gaze (and, accordingly, their attention) to the next stimulus as soon as processing the currently fixated one is sufficiently advanced^58^. Second, this technique abandons the need for an additional (“surrogate”) task which could have imposed processing demands beyond mere word recognition. Expectedly, our participants exhibited longer first fixations and longer gaze durations on pseudowords than on words which proves that the participants processed the stimuli. At the neural level, the challenging aspect of the technique is that the “events” for modeling the haemodynamic response function (i.e., the onsets of fixations) occur in rapid succession because of the brevity of a typical fixation (see Ref. 12 for an in-depth discussion). To illustrate, fixations during reading last typically 250 ms and are separated by very brief saccades (~30 ms)^31^. On the upside, the technique brings in the advantage of eye movement studies, that is, one can – in a similar timeframe – administer a much higher number of “trials” than in conventional experimental tasks. The reasons are the short (but sufficiently long) “presentation durations” of the items (if fixation times are equated with presentation duration) and the fact that the “interstimulus intervals” for the simultaneously presented items are exceptionally short. (The processing of the items is only segregated by saccades during which visual information uptake is suppressed^59^). In a previous study from our lab^13^, we provided the proof-of-concept that fixation-related fMRI is, despite the rapid succession of events, sensitive enough to reveal the differences in neural activation for reading material (words and pseudowords) compared to visual control stimuli (e.g., strings of unfamiliar letters; see Introduction). Similar to our previous study, the reading material of the present study activated the well-established, left-lateralized reading network^4^,^5^,^6^,^7^,^8^. More specifically, alongside with the activation in the left vOT, we observed activation in the left middle and superior temporal gyrus and in left inferior frontal gyrus.

### Conclusion and future direction

Our finding of higher activation for words than pseudowords in the left vOT was obtained with the novel fixation-related fMRI technique which made a surrogate “reading” task unnecessary. The finding suggests that the higher activation for pseudowords, which is predominantly reported in the literature, may in fact be a consequence of task properties. Our results are, thus, hard to reconcile with the assumption of the Interactive Account about automatically generated predictions which in case of pseudowords should have led to an elevated prediction error and hence to higher left vOT activation compared to words. For future studies, fixation-related fMRI offers the possibility of assessing brain activity while participants silently read whole sentences or short paragraphs. Thus, investigating the neural correlates of predictive coding during natural reading is a venue for future research.

## Additional Information

**How to cite this article**: Schuster, S. *et al.* Eyes on words: A fixation-related fMRI study of the left occipito-temporal cortex during self-paced silent reading of words and pseudowords. *Sci. Rep.* **5**, 12686; doi: 10.1038/srep12686 (2015).

## Figures and Tables

**Table 1:** Means and standard errors (in parenthesis) of the eye-tracking results.

	Words	Pseudowords	Slashes
Number of fixations	1.34 (0.03)	1.39 (0.04)	1.25 (0.03)
First fixation duration [ms]	295 (10)	311 (13)	319 (13)
Gaze duration^a^ [ms]	356 (14)	383 (17)	376 (18)

*Note*.

^a^Gaze duration is defined as the sum of all fixation durations during the first encounter of a word (excluding regressions).

**Table 2:** Brain regions significantly activated by word and pseudoword reading compared to slash-string scanning.

Region	MNI coordinates			*t*	Voxel extent
	x	y	z		
*Words* > *Slashes*					
L Fusiform Gyrus	−39	−46	−20	8.54	428
L Middle Temporal Gyrus	−60	−31	−2	6.06	223
L Precentral Gyrus	−54	−4	43	5.31	233
R Inferior Occipital Gyrus	36	−91	−11	5.15	48
L Inferior Frontal Gyrus (orbital part)	−36	29	−5	4.74	101
*Pseudowords* > *Slashes*					
L Fusiform Gyrus	−42	−52	−23	5.94	226
L Superior Temporal Gyrus	−66	−40	4	5.88	84
L Precentral Gyrus	−51	−7	40	4.86	42

*Note*. R = right; L = left
